# The evolutionary history of histone H3 suggests a deep eukaryotic root of chromatin modifying mechanisms

**DOI:** 10.1186/1471-2148-10-259

**Published:** 2010-08-25

**Authors:** Jan Postberg, Sakeh Forcob, Wei-Jen Chang, Hans J Lipps

**Affiliations:** 1 Institute of Cell Biology, University of Witten/Herdecke, Witten, Germany; 2 Department of Biology, Hamilton College, Hamilton, NY, USA

## Abstract

**Background:**

The phenotype of an organism is an outcome of both its genotype, encoding the primary sequence of proteins, and the developmental orchestration of gene expression. The substrate of gene expression in eukaryotes is the chromatin, whose fundamental units are nucleosomes composed of DNA wrapped around each two of the core histone types H2A, H2B, H3 and H4. Key regulatory steps involved in the determination of chromatin conformations are posttranslational modifications (PTM) at histone tails as well as the assembly of histone variants into nucleosomal arrays. Although the mechanistic background is fragmentary understood, it appears that the chromatin signature of metazoan cell types is inheritable over generations. Even less understood is the conservation of epigenetic mechanisms among eukaryotes and their origins.

**Results:**

In the light of recent progress in understanding the tree of eukaryotic life we discovered the origin of histone H3 by phylogenetic analyses of variants from all supergroups, which allowed the reconstruction of ancestral states. We found that H3 variants evolved frequently but independently within related species of almost all eukaryotic supergroups. Interestingly, we found all core histone types encoded in the genome of a basal dinoflagellate and H3 variants in two other species, although is was reported that dinoflagellate chromatin is not organized into nucleosomes.

Most probably one or more animal/nuclearid H3.3-like variants gave rise to H3 variants of all opisthokonts (animals, choanozoa, fungi, nuclearids, Amoebozoa). H3.2 and H3.1 as well as H3.1t are derivatives of H3.3, whereas H3.2 evolved already in early branching animals, such as *Trichoplax*. H3.1 and H3.1t are probably restricted to mammals.

We deduced a model for protoH3 of the last eukaryotic common ancestor (LECA) confirming a remarkable degree of sequence conservation in comparison to canonical human H3.1. We found evidence that multiple PTMs are conserved even in putatively early branching eukaryotic taxa (Euglenozoa/Excavata).

**Conclusions:**

At least a basal repertoire of chromatin modifying mechanisms appears to share old common ancestry and may thus be inherent to all eukaryotes. We speculate that epigenetic principles responsive to environmental triggers may have had influenced phenotypic variation and concomitantly may potentially have had impact on eukaryotic diversification.

## Background

**Figure 1:**
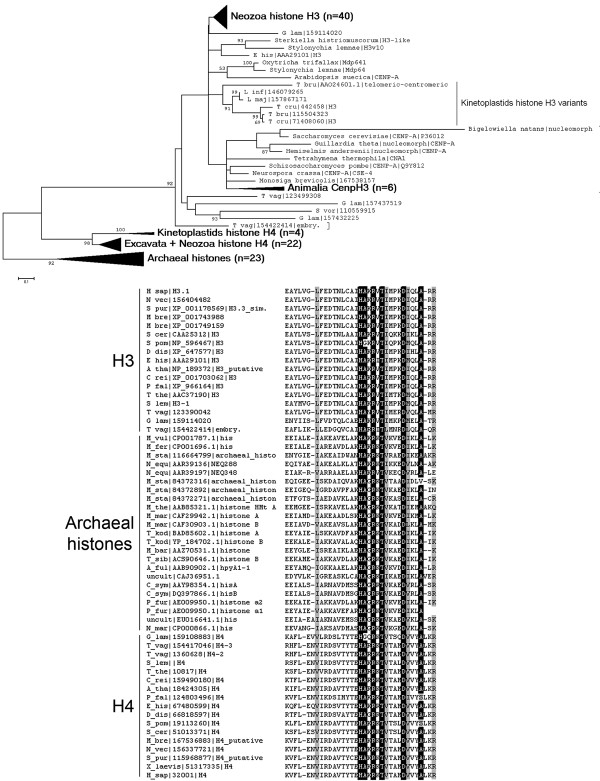
**Phylogenetic relationsship between eukaryotic core histone types H3 and H4 as well as archaeal histones**. A bootstrap consensus Neighbour Joining tree (A.) illustrates the phylogenetic relationsship between eukaryotic histones H3 and H4 as well as archaeal histones, which share common ancestry. More divergent H3 and H4 variants of kinetoplastids occur as sister groups with regard to their variants from other eukaryotes, probably due to long branch attraction. Similarly CenH3 variants occur as long branching sequences. The protein sequence alignment (B.) shows a conserved region from the histone fold domains of several eukaryotic histones H3 and H4 as well as archaeal histones. Residues identical in >95% of all sequences are shaded black. Residues similar in >95% of all sequences are shaded grey.

The regulation of eukaryotic gene expression occurs in the context of DNA fibres compacted by interactions with proteins - the chromatin, where on the first level of compaction the DNA is wrapped around a protein octamer composed of the four core histone proteins types H2A, H2B, H3 and H4 forming the nucleosome. This fibre becomes further compacted by the interaction with linker histone H1 and other proteins, forming a 30 nm fibre [1,2]. Many archaea are known to possess histones, which most probably share common ancestry with the histone fold domains of eukaryotic H3 and H4. Short conserved segments (corresponding to human H3.1E98-R130 or human H4K60-R93, respectively) shared by many archaeal histones and eukaryotic H3 and H4 are illustrated in Figure 1. These archaeal histones interact with the genomic DNA as tetrameric complexes [3-5]. In eukaryotes posttranslational modifications (PTMs, e.g. acetylation, methylation, phosphorylation) at the N-termini of all histone types can alter the degree of chromatin compaction [6]. Besides PTMs the incorporation of histone variants into chromatin, particularly of histones H3 and H2A, seems to play a crucial role for the establishment of specific chromatin states. Histone H3 has a globular C-terminal domain (histone fold), which harbours four helix motifs (αN, α1, α2, α3). A putative recognition site for histone chaperones involved in nucleosome assembly partially overlaps with the α2-helix (compare Additional file 1). Most PTMs are targeted to the unstructured N-terminus that protrudes from the nucleosome [7-9]. It seems to be a general eukaryotic feature that the unstructured N-terminal sequences of H3 are more divergent between species than the C-terminal globular domain.

Chromatin modifying mechanisms play a superordinate role in the orchestration of developmental processes, thus influencing phenotypic differentiation. Recent studies have also shown that some chromatin modifying mechanisms are responsive to environmental agents [10-14], which hereby can act as triggers of gene expression. It is under controversial discussion whether some epigenetic features, which include PTMs of histones and assembly of specific histone variants into discrete chromatin segments, contribute to epigenetic memory and may thus be inheritable over generations without genotypic changes [6,9,15]. Accordingly epigenetic mechanisms possibly may affect natural selection and thus had influenced the diversification of eukaryotic life [16].

However, the diversity of epigenetic mechanisms, their common themes as well as differences, and finally their potential impact to the evolution of eukaryotes remains largely unexplored. From all core histone types, variants of histone H3 with their dynamic posttranslational modifications have been most extensively studied in selected model organisms to date. In order to discover the evolutionary history of basal chromatin modifying mechanisms, we decided to undertake combined phylogenetic and molecular biological analyses focusing on variants of the core histone H3 and its PTM signature.

## Results and Discussion

To date phylogenetic analyses of histone H3 variants were limited due to poor data availability of sequences from species representing putatively early branching eukaryotes. If not neglected totally parasitic organisms, such as *Entamoeba*, were usually used to represent putatively early branching eukaryotic supergroups [17-19]. On the other hand multicellular organisms were often overrepresented in such analyses. The highly divergent H3 variants of some parasitic organisms usually led them to be placed at the basis of phylogenetic trees, whereas histone H3 family members of metazoa and plants often appeared as "crown group" members. Although it was already known for decades that histone H3 is highly conserved in many eukaryotic species, the topology of such phylogenetic trees could be interpreted in a way that the ancestral eukaryotic histone H3 was highly divergent in comparison with H3 of "crown group" eukaryotes. However, we hypothesize that the placement of the divergent H3 variants of parasites near the root of such trees was an artifact due to long-branch attraction.

We therefore reinvestigated the phylogeny of histone H3 in the light of recent progress in understanding the tree of life and eukaryogenesis [20-24]. While some uncertainty about the position of the eukaryotic root remains (whether it is 1. between unikonts and bikonts, 2. inside the excavates, or 3. between early diverging euglenozoa and excavates), it appears that opisthokonts (animals, choanozoa, fungi) and amoebozoa (all grouped together as Unikonta) diverged early from chromists and plants (part of the Bikonta group), resulting in a deep cleft between those eukaryotic supergroups and a multifurcated tree without "crown groups" (Additional file 2). Importantly, we found that the protein sequences of some histone H3 variants between selected species from Unikonta and Bikonta are remarkably invariant. To mention only two of multiple examples, histone H3 variant protein sequences between the choanoflagellate *Monosiga brevicollis *(Unikonta; XP_001749159) and *Arabidopsis thaliana *(Bikonta; NP_189372) vary in only 2 out of 135 residues (~98,5% identity). Similarly, histone H3 from *Nuclearia simplex *(Unikonta; NXL00000490) deviates in only 4 out of 135 residues from histone H3 in the green alga *Ostreococcus lucimarinus *(Bikonta; ABO96363) (~97% identity) (Additional file 1; Additional file 2; Additional file 3). Histone H3 variants ancestral to choanoflagellates and plants or nuclearids and green algae, respectively, consequently had most likely been very similar to these histone H3 variants. As a working hypothesis we therefore assumed, that this possibly could even be true for the H3 (variants) of the last eukaryotic common ancestor (LECA).

### Highly conserved H3 variants occur even in putatively early branching eukaryotic clades

To test our working hypothesis we resampled histone H3 protein sequences from all eukaryotic supergroups (Opisthokonta, Amoebozoa, Archaeplastida, Rhizaria, Chromalveolata and Excavata) [25] using various databases from completely sequenced genomes or fragmentary EST projects as sources (Histone Sequence Database: http://genome.nhgri.nih.gov/histones/; GeneBank: http://www.ncbi.nlm.nih.gov/Genbank/; RefSeq: http://www.ncbi.nlm.nih.gov/RefSeq/; TBestDB: http://tbestdb.bcm.umontreal.ca/searches/welcome.php). We focused on the identification of H3 sequences from non-parasitic organisms representing putatively early branching Euglenozoa and Excavata. Importantly, we were able to assemble multiple new histone H3 sequences from various species of putatively early branching eukaryotes (among others: *Reclinomonas americana*, *Euglena gracilis*, *Naegleria gruberi*, *Sawyeria marylandensis*, *Streblomastix strix*). We also obtained sequences of histone H3 variants from a ciliated protist, *Stylonychia lemnae *(Additional file 1; Additional file 3). To our best knowledge the resulting dataset represents the most complete representation of histone H3 variant sequences available.

Interestingly, we found extremely conserved histone H3 sequences and remarkable examples of divergent H3 variants in all eukaryotic supergroups (Additional file 1; Additional file 3), importantly also in non-parasitic Euglenozoa and Excavata. Presuming an early divergence of Unikonta and the Plantae/Chromista groups from Excavata or Euglenozoa, respectively, we assumed that the protoeukaryotic histone H3 (protoH3) of LECA must have been rather invariant from canonical histone H3 (human H3.1).

**Figure 2:**
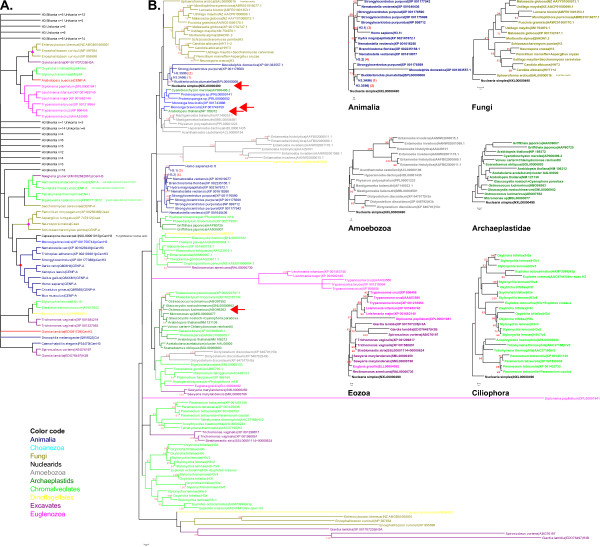
**The evolutionary history of histone H3 and CenH3 variants**. A. The evolutionary history of 159 H3 and CenH3 variants was inferred using the Neighbor-Joining method [48]. B. The evolutionary relationship of 128 non-redundant histone variants was inferred using the Neighbor-Joining method [48]. Importantly, animal stem H3 variants are identical in a broad range of species: For example, H3.3A96 (1) is identical in *Trichoplax*, *Hydra*, *Nematostella*, *Buddenbrockia*, and identical H3.3S96 (2) is found in *Drosophila*, *Strongylocentrotus*, *Branchiostoma*, *Xenopus *and many mammals. Further, H3.1 (3) is identical in mammals from mouse to human. Identical H3.2 (4) variants occur in organisms like *Trichoplax*, *Drosophila*, *Branchiostoma*, *Xenopus *and many mammals. The monophyly of several eukaryotic clades was well supported by phylogenetic analyses of histone H3 variant sequences. Pairwise comparison of selected H3 variants (indicated by arrows) from Unikonta or Bikonta species, respectively, revealed very high degrees of sequence conservation resulting in only rough separation of these clades. Due to very limited sequence variability no support for chromista or plant monophlyly could be found. However, two ciliate classes, Oligohymenophorea (e.g. *Tetrahymena *and *Paramecium*) and Spirotrichea (e.g. *Stylonychia *and *Euplotes*) were faithfully separated. Importantly, multiple H3 variants from Eozoa (Excavata + Euglenozoa) branched close to conserved H3 variants from other groups, predominantly Chromalveolata (*Euglena*, *Reclinomonas*, *Sawyeria*, *Trichomonas*, *Streblomastix*). All long branching Eozoa (*Leishmania*, *Trypanosoma*, *Diplonema*, *Giardia*, *Spironucleus*) or Microsporidia (*Enterocytozoon*, *Encephalitozoon*) H3 variants are parasites.

To strengthen this hypothesis we performed phylogenetic analyses using a combined histone fold dataset of H3 variants as well as CenH3 variants. These analyses typically resulted in tree topologies, where putative stem H3 variants occurred separated from divergent parasitic H3 variants (e.g. from Kinetoplastids or *Giardia lamblia*) as well as CenH3 variants (Figure 2A). A common ancestry of mammalian and avian CENP-A proteins (to some extent also of lower vertebrate and non-vertebrate CENP-As; clade marked by red rhomb in Figure 2A) was supported by high bootstrap values. Further a fungal CenpH3 clade was well supported (clade marked by magenta rhomb in Figure 2A). However, since divergent H3 variants and other eukaryotic CenH3 s did not occur as monophyletic groups, proteins not characterized yet could not be assigned to a H3-like or CenH3-like function by their phylogenetic position. Our analyses leave open whether a protoCenH3 ancestral to all eukaryotic CenH3 s had existed or whether extant CenH3 s have multiple origins in eukaryotic evolution.

We next inferred H3 variant phylogenetic trees using a dataset of full-length protein sequences from which long branching H3 variants with uncertain position were eliminated (Figure 2B; Additional file 1; Additional file 3). Such sequences mostly represented parasitic species (among others *Encephalitozoon*, *Giardia*, *Spironucleus*), which showed tendency to be positioned at the bottom of trees, including all characterized and putative CenH3 derivatives. Although the bootstrap support for such unrooted trees was weak for numerous clades, the monophyly of many groups was well represented (e.g. Animals, Fungi, Oligohymenophorea/Ciliophora/Chromista, Spirotrichea/Ciliophora/Chromista), whereas the monophyly of Amoebozoa, Chromista (Apicomplexa, Heterokonta, Rhizaria) and Archaeplastidae could only weakly or not be resolved, probably due to the generally very high degree of H3 sequence similarity in those groups (Additional file 1; Additional file 3). As a global observation we discovered, that variations in H3 protein sequences often occurred within motifs involved in writing and reading the PTM signature as well as in the putative histone chaperone recognition domain (amino acids 85-101, referring to human H3.1).

### Histone H3 variants have evolved frequently, but independently in related species of almost all eukaryotic supergroups

Differences between H3 variants recognized frequently involve the presence or absence of discrete putative phosphorylation sites (e.g. S/N10, S/T/A28, S/T/A31, S/A96), suggesting a cell cycle dependent regulation by phosphorylation of specific H3 variants. Since phosphorylation of serine, or presumably also threonine, can prevent or even disrupt binding of effector proteins (e.g. heterochromatin-binding protein 1, HP1) at adjacent methylated lysine residues, it can be assumed that such sites could also function as switches regulating the chromatin signature [26,27]. The presence or absence of phosphorylation sites therefore suggests important non-redundant biological functions of such H3 variants.

Our data suggest, that a variant similar to the replication-independent mammalian histone H3.3 - but not the (canonical) H3.1 - was likely ancestral to H3 variants of fungi and their sister group nuclearids as well as choanozoa and animals. In many metazoan species H3.3 occurs with identical (e.g. in human, mouse, *Xenopus*, *Branchiostoma*, *Drosophila*) or slightly different protein sequence (S96 replaced by A96 in *Hydra*, *Nematostella*, *Trichoplax*). Notably, in our phylogenetic analyses the only H3 from *Nuclearia simpex *we could identify occurs near the root of the animal H3.3 clade. This H3.3-like variant deviates from human H3.3 only in one substitution (H3.3S87 in *Nuclearia*; H3.3A87 in *Homo*). In Opisthokonta S87 occurs predominantly in fungal H3 variants (notably also in animal H3.2 and H3.1), whereas A87 is found in animals, choanoflagellates and most sequences of Amoebozoa. For example in two putative stem Amoebozoa species A87 is found in a H3 variant of *Mastigamoeba balamuthi *(~94% identical to animal/nuclearid H3.3), whereas S87 is found in a 118 aa H3 sequence fragment of *Hyperamoeba dachnya *(~90% identical). Remarkably, S87 dominates in H3 variants of Bikonta. We conclude that one or more H3 variants very similar to animal/nuclearid H3.3 gave rise to all H3 variants found in extant opisthokonts.

Deriving from an animal/nuclearid H3.3S87-like precursor, we found identical homologs of histone H3.2 in early branching animals, such as *Trichoplax adherens*, suggesting that this replication-dependent H3 variant might have evolved early during metazoan evolution, as well as in organisms like *Drosophila*, *Branchiostoma*, *Xenopus*, *Monodelphis*, mouse and human. H3.1, which only differs from H3.2 insofar that H3S96 is replaced by H3C96, as well as the testis-specific H3.1t could only be found in mammals. These H3 variants most likely have a late origin in metazoan evolution. Putative additional variants were also identified in some animals (Additional file 1; Additional file 3).

**Figure 3:**
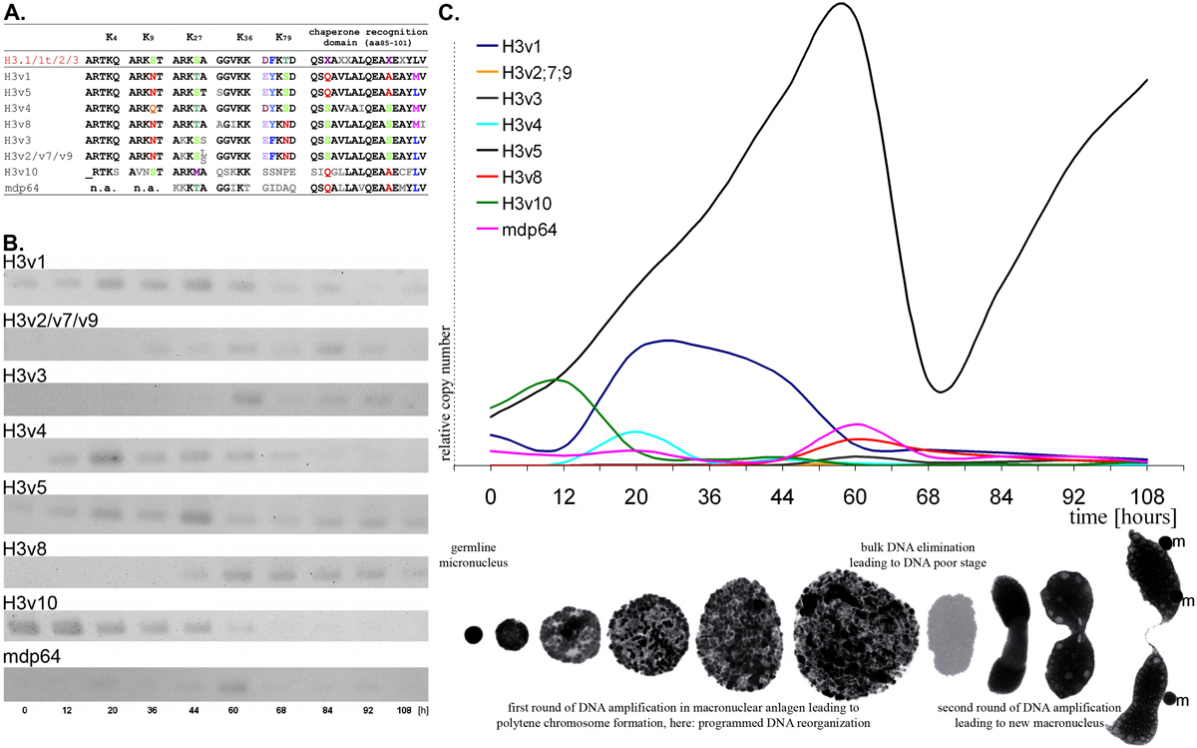
**Numerous histone H3 variants are differentially expressed in the course of macronuclear differentiation in *Stylonychia lemnae***. A. Conservation of sequence motifs adjacent to N-terminal lysine residues and chaperone recognition sites in human H3.1 (top line) and *Stylonychia histone *H3 variants. The descending order of *Stylonychia *H3 variants reflects the phylogenetic distance compared to human H3.1. B. Agarose gel electrophoresis of PCR products amplified from developmental stage-specific cDNA. C. Quantitative Real Time PCR analysis of *Stylonychia *H3 variants expression. The morphology of developing macronuclei - revealed by confocal laser scanning microscopy of To-Pro-3 stained DNA - at successive stages is shown in relation to the time scale (hours post conjugation).

Interestingly, in animals the highest number of H3 variants was identified in the sea urchin *Strongylocentrotus purpuratus *(5)* (*Numerical data displayed here and below exclude long branching H3 variants, whose biological function could deviate from "nucleosomal" H3 s, as well as CenH3s). However, the occurrence of numerous H3 variants is not restricted to animals. Our analyses strongly suggest that they have evolved frequently and independently in many eukaryotic taxa. For example, we characterized the macronuclear genomic sequences of at least 7 core histone H3 variants and one putative CenH3 variant (mdp64) in the spirotrichous ciliate species *Stylonychia lemnae*, which have been partially identified before by Bernhard [28] - to our best knowledge the highest number of H3 variants found so far in nature. Interestingly, the main differences of these *Stylonychia *H3 variants are within sequence motifs known to be involved in 'writing' or 'reading' the histone PTM signature, thus determining chromatin higher order structure (Figure 3A; Additional file 1; Additional file 3). Since spirotrichous ciliates like *Stylonychia *exhibit enormous developmental reorganization of their genome during sexual reproduction, involving multiple epigenetic mechanisms [29-31], it can be speculated that those H3 variants could play important roles in the regulation of these processes. To address this hypothesis experimentally we performed expression analyses of *Stylonychia lemnae *histone H3 variants by PCR (Figure 3B) and quantitative real-time PCR (Figure 3C) using developmental stage specific cDNA. Notably, we were not able to faithfully distinguish the highly similar variants H3v2, H3v7 and H3v9. We therefore decided to treat H3v2, H3v7 and H3v9 as equal. These experiments not only demonstrated that all H3 variants were expressed in a developmental stage specific manner, but also showed significant differences in their relative expression rates (e.g. on their peaks of expression high levels of H3v5, H3v1 and H3v10 could be detected, while relative levels of mdp64, H3v4, H3v8, H3v3 and H3v2/v7/v9 were lower). Interestingly, the expression of some variants (H3v1, H3v4, H3v10) was pronounced during the first round of DNA amplification in the course of macronuclear differentiation, while other variants were expressed at the onset of, or during the second round of DNA amplification (H3v2/v7/v9, H3v3, H3v8, mdp64). The expression level of H3v5 appeared to increase or decrease in parallel to the DNA content in macronuclear anlagen, respectively (compare [29]). Although detailed experimental data about the biological relevance of each particular H3 variant in *Stylonychia *is not yet available, their differential expression strongly suggests that they are functionally non-redundant. Theoretically it can be ruled out to some extent that at least a sub-fraction of the H3 variant nanochromosomes encode non-functional proteins or represent pseudogenes, since programmed DNA reorganization in spirotrichous ciliates involves a comparison between the germline (micronuclear) and the somatic (macronuclear) genomes resulting in a selection of macronucleus-destined sequences (reviewed in [30]). In spirotrichous ciliates this genome comparison apparently involves a proof-reading template from the old macronucleus [32], which can be RNA [33]. We assume that such a proof-reading mechanism could generally and efficiently lead to the elimination of non-expressed nanochromosomes from the developing macronucleus.

Examples of increased H3 variant numbers within related species could be found in almost all eukaryotic supergroups by our analyses (Additional file 1; Additional file 3) or by other researchers (compare public databases mentioned above), for example in Fungi (3 in *Candida albicans*), Amoebozoa (3 in *Entamoeba sp*. and *Dictyostelium discoideum*), Plants (3 in *Arabidopsis thaliana*), Apicomplexa/Chromista (3 in *Plasmodium falciparum*), Heterokonta/Chromista (2 in *Hyaloperonospora parasiticum*, *Phaeodactylum tricornutum*, *Phytophtora infestans*), also in excavates (2 in *Sawyeria marylandensis*, *Trichomonas vaginals*) and more divergent H3 variants in euglenozoa (2 in *Trypanosoma sp*.). Since we could identify only one H3 sequence from a Rhizaria species, we can make no statement for this group, whether H3 variants exist or not.

### Do derived H3 variants exist in dinoflagellates?

Using nuclease digestion experiments and electron microscopic approaches it has been observed, that the most portion of chromatin in dinoflagellates is not organized into nucleosomal repeats [34-36]. In all dinoflagellates examined to date the DNA:protein ratio within chromatin is very small (~10:1). The protein fraction contains several basic histone-like proteins, which exhibit some similarities with both bacterial histone-like proteins and eukaryotic histone H1 [37]. Surprisingly, we found putative H3 variants in *Perkinsus marinus *(inc. sed., probably related to dinoflagellates or apicomplexans) as well as in the dinoflagellates *Pyrocystis lunula *and *Karlodinium micrum *(Additional file 1; Additional file 3). Histone H3 of *Perkinsus marinus *possesses several conserved motifs adjacent to lysines targeted by PTM in other eukaryotes (e.g. K4, K9, K27, K36), whereas K9/K27 motifs are absent in *Karlodinium micrum *and *Pyrocystis lunula*. Furthermore the K36 motif lacks in *Pyrocystis lunula*. Since the *Pyrocystis *H3 variant seems to be among numerous genes whose expression profiles are affected by oxidative stress [38], evidence exist at least for this H3 variant at both the genomic and the transcriptional level.

Remarkably, in *Perkinsus marinus *we found also sequences encoding core histones H4 (GeneBank XM_002777579), H2A (GeneBank XM_002772145) and H2B (GeneBank XM_002787339) but no sequences homologous to other dinoflagellate histone-like proteins [37], suggesting that the chromatin organization of this basal dinoflagellate [39] relies on nucleosomes.

On closer look we could not identify further core histone types in the genomes of *Karlodinium micrum *or *Pyrocystis lunula*, whereas we found one H2A-family sequence fragment in another dinoflagellate, *Alexandrium tamarense *(GeneBank AY849372). Our observation suggests that these histone variants are the only core histone types involved in chromatin organization in these species, raising the question what could be the consequences on chromatin structure. Without experimental evidence we can only speculate that in dinoflagellates like *Karlodinium micrum *or *Pyrocystis lunula*, which apparently do not possess a complete set of core histone types, H3 variants could be involved into chromatin organization of a fraction of the genome, similar to spermatozoa in many species, which replace most histones by protamines but retain nucleosomal chromatin organization at some genomic loci (reviewed in [40]). It cannot be excluded that H3 variants interact with histone-like proteins. But realizing that the histone-fold domains of all four core histones are structurally very similar [8], it also seems very plausible that the formation of H3 homodimers which possibly further assemble into tetramers and octamers is propagated.

However, the presence of all four core histone types in a basal dinoflagellate like *Perkinsus marinus *and also the evidence for single core histone types in other species strongly support the view that the alternative chromatin organization in most representatives of that phylum is a derived, not an ancestral feature in dinoflagellate chromatin evolution.

### Divergent histone H3 variants from various supergroups represent a derived, not the ancestral state

**Figure 4:**
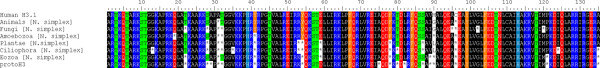
**Reconstruction of ancestral histone H3 states**. The ancestral state reconstruction of histone H3 variants from various clades (corresponding nodes are highlighted in the group-specific phylogenetic trees by red rhombs in Figure 2B) and H3 variant(s) of LECA confirms a high degree of sequence identity or similarity, respectively (shaded columns). Variable sites are highlighted (*); color scheme: basic amino acids (blue), acidic amino acids (red), aromatic amino acids (orange), putative phosphorylation sites (green). Nuclearia simplex H3 was used as outgroup for all group-specific trees. A detailed overview about the most frequent residues observed at such variable site is given in Additional file 4.

Due to the presence of conserved H3 variants with high similarity to animal/nuclearid H3.3-like variants in selected species of all eukaryotic supergroups, it may be concluded that ancestral states are unlikely to be represented by the more divergent H3 variants in various supergroups. Following these assumptions we deduced ancestral states for selected clades well supported in our phylogenetic analyses and subsequently a putative protoH3 sequence, which exhibits 87% sequence identity compared with human (canonical) H3.1 (Figure 4; Additional file 4), confirming our initial hypothesis, that protoH3 variant(s) of LECA were rather invariant from extant stem H3-like variants (e.g. animal or nuclearid H3).

**Figure 5:**
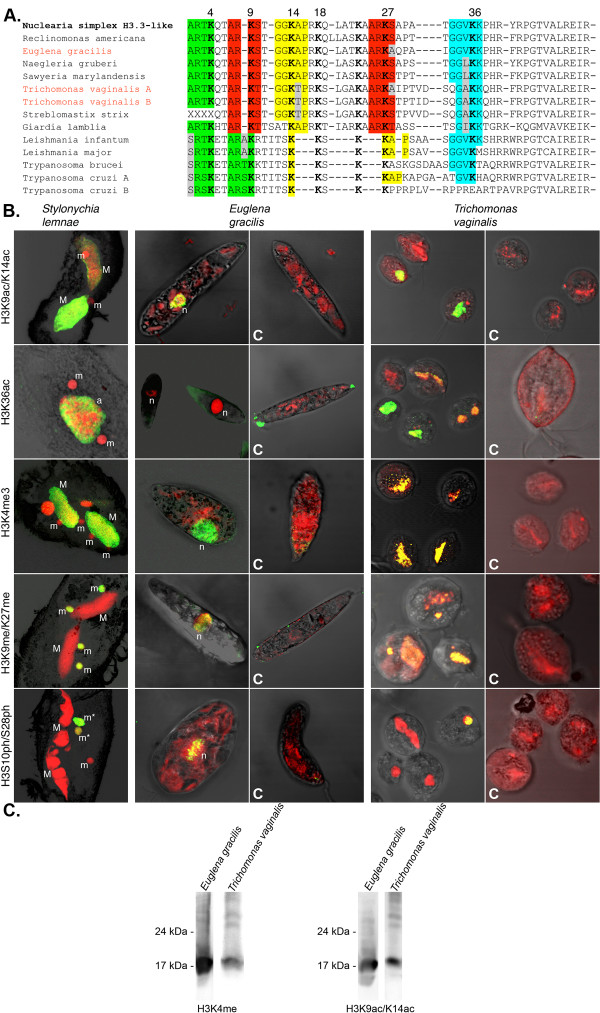
**Multiple histone H3 modifications are conserved in putatively early branching eukaryotes**. Posttranslational histone H3 modifications (PTM) occur at conserved N-terminal sequence motifs (color shaded in A.) in *Euglena gracilis *as well as *Trichomonas vaginalis *as suggested by immunofluorescence (B.) and Western (C.) analyses. C-marked images represent peptide competition assays as antibody specificity control. Both species represent putatively early branching eukaryotic clades. In the immunofluorescence panel (B.) the various PTMs occur as green signals, whereas nuclei and in some cases other nucleic acid-containing structures occur as red signals. In some cases DNA containing structures where labelled as follows: micronucleus/during mitosis (m/m*), macronucleus (M), nucleus (n). Western analyses (C.) confirm that the antibody targeted to H3K4me3 reacts with a protein band similar in size to histone H3 in *Euglena *and *Trichomonas*. Even H3K9ac/K14ac was detected in both *Euglena *and *Trichomonas*.

Among the variable residues, three positions (2%) alter between the aromatic amino acids tyrosine (Y) or phenylalanine (F). The observed presence or absence of putative phosphorylation sites at various positions as a character state of many histone H3 variants is nicely confirmed by the reconstruction of ancestral H3 s of all supergroups as well as LECA's protoH3 (Figure 4; Additional file 4). It seems therefore reasonable to assume that multiple H3 variants could have had already evolved in LECA. Importantly, almost all lysines (K) are conserved in the reconstructed ancestral H3 sequences, with the notable deviation of K54 from euglenozoa and excavates, which alters between K and R (arginine) in chromists and plants and has evolved to R54 in opisthokonts and amoebozoa (Figure 4; Additional file 4). Interestingly, the number of N-terminal lysines in the divergent H3 variants of *Trypanosoma *and *Leishmania *is almost identical to canonical H3 (compare Figure 5A; Additional file 1; Additional file 3).

### At least a basal repertoir of histone H3 modyfing mechanisms shares common ancestry in all eukaryotes

As a consequence of this significant invariance of histone H3 in the course of eukaryotic evolution, where diverging H3 variants reflect a derived - not the ancestral state, conserved epigenetic mechanisms targeting histone H3 N-termini could be more widespread among eukaryotes than expected or even primarily inherent to all eukaryotes. To test whether selected PTMs occur at conserved H3 N-termini (Figure 5A) in H3 variants of presumably early branching eukaryotes, we performed immunofluorescence microscopy (Figure 5B) and in some cases Western analyses (Figure 5C) using antibodies targeted to specific histone modifications, which tolerate slight alterations in adjacent amino acid motifs but are reported to faithfully recognize the respective PTM. We selected *Euglena gracilis *(Euglenozoa/non-parasitic) and *Trichomonas vaginalis *(Excavata/parasitic) as representative species, since *Euglena *histone H3 differs in only one amino acid (A28) from human H3.1 (S28) in the N-terminal 40 residues (~98% identity). Although the N-terminal sequence identity in comparison with human H3.1 does not exceed ~78% in two H3 variants of *Trichomonas vaginalis *the overall similarity of these variants is higher than in other parasitic Excavata/Euglenozoa model systems, such as *Giardia*, *Leishmania *or *Trypanosoma*. Moreover motifs adjacent to K4, K9, K14, K27 and K36 exhibit a high degree of conservation. Importantly, we performed competition assays as controls as described in [29]. For comparison we monitored PTMs in various nuclear types of the ciliate *Stylonychia lemnae*, since antibodies used have been extensively tested in this single cell organism before [29]. We found multiple examples of PTMs occurring in nuclei of both species, *Euglena gracilis *as well as *Trichomonas vaginalis*. Histone H3 acetylation at K9 or K14, which in *Stylonychia *occurs in the transcriptionally highly active macronuclei (M), was detected in nuclei (n) of *Euglena *and in most undistinguished stages observed in *Trichomonas*. Using antibodies targeted to H3K36ac, which in *Stylonychia *is restricted to developing macronuclear anlagen (a), we could not detect this PTM in *Euglena*, whereas signals were prominent in nuclei of most stages of *Trichomonas*. H3K4me3 is mostly associated with transcriptional activity as highlighted by strong macronuclear signals in *Stylonychia *macronuclei (M). This PTM was detected in nuclei (n) of *Euglena *and many undistinguished stages of *Trichomonas*. H3 methylated at K9 or K27 in the context of ARKS/T sequence motifs frequently propagates binding of heterochromatin binding protein 1 (HP1)-like chromobox proteins, often resulting in heterochromatin formation. In *Stylonychia *H3K27me3 occurs in the heterochromatic micronuclei (m), which are silent in gene expression. Using an antibody which cross-reacts with H3K9me3 and H3K27me3 we observed nuclear signals corresponding to ARKme3S/T in both *Euglena *and *Trichomonas*. The assumption that ARKme3S/T within H3 tails could be involved in heterochromatin formation even in early branching eukaryotes is strengthened by the presence of numerous HP1-like chromodomain proteins encoded in the genomes of several representative species, with at least 8 chromodomain proteins being encoded in the genome of *Trichomonas vaginalis *(Additional file 5). Chromodomain sequences of exemplary HP1 homologs shown in Additional file 5 have formally the ability to bind ARKme3S/T, as it might carefully be concluded from the conservation of 3 aromatic residues forming an "aromatic cage" in HP1, which seems to be crucial for ARKme3S/T binding [41,42]. At least a subfraction of these proteins possess the typical HP1-like domain organization with a N-terminal chromodomain, which usually contributes to the binding of ARKme3S/T, and a C-terminal chromoshadowdomain, which is thought to be involved in heterochromatin spreading (Daniele Canzio, Narlikar Lab, submitted, pers. communication). Besides other biological functions, the formation of condensed chromosomes during mitosis involves H3S10ph and/or H3S28ph in many organisms, as shown here for H3S28ph during micronuclear division (m*) in *Stylonychia*. We observed that H3S10ph also occurred in nuclei of *Euglena *with a signal distribution reminiscent of condensed chromosomes, suggesting that this biological function may have a deep eukaryotic root. Remarkably, the only *Euglena *H3 variant recognized does not posses H3S28. Occasionally, H3S10ph or H3S28ph was also observed in nuclei of *Trichomonas*. Western analyses using antibodies targeted to H3K4me3 or H3K9acK14ac revealed a H3-sized protein band (Figure 5C), whereas other antibodies used in microscopy failed to detect linearized proteins immobilized on a nitrocellulose membrane.

Remarkably, multiple PTMs at specific sites have also been identified in the divergent core histone types of *Trypanosoma brucei *[43,44]. Thus our analyses contribute to the view that numerous PTMs occur in various Excavata and Euglenozoa. With regard to the very high degree of H3 protein sequence similarity and multiple conserved PTMs found, especially in non-parasitic *Euglena gracilis*, it seems likely that major chromatin modifying mechanisms evolved early during eukaryogenesis, possibly directly accompanying the acquisition of the nucleus, the invasive accumulation of genomic non-coding DNA and the organization of the genome into chromosomes. We therefore speculate that such conserved epigenetic mechanisms, if inheritable, may have had substantially contributed to the adaptation of organisms to environmental changes and consequently to the diversification of eukaryotic life. However, conflicting with this speculation, the very basic problem remains unsolved, whether a genomic feedback on epigenetic manifestations leading to genome encoded epigenetic signatures is obligatory, or whether a long-term genome-independent persistence of epigenetic signatures over many generations exists.

## Conclusions

Multiple histone H3 variants evolved frequently but independently within related species of almost all eukaryotic supergroups, whereby the presence of numerous variants in Rhizaria could not be shown due to the limited sequence data. Remarkably, we found at least 7 histone H3 variants in the spirotrichous ciliate *Stylonychia lemnae*, which are expressed in a developmental stage specific manner, but also show significant differences in their relative expression rates.

Interestingly, although it has been reported that dinoflagellate chromatin is not organized into nucleosomes, we found that the genome of the basal dinoflagellate species *Perkinsus marinus *encodes all four core histone types suggesting a "classical" nucleosome-based type of chromatin organization. Moreover we found H3 variants encoded in the genomes of at least two other dinoflagellate species. A recurring theme in variants of histone H3 in almost all eukaryotic supergroups is the presence or absence, respectively, of discrete putative phorsphorylation sites.

Our data confirm that an animal/nuclearid-like histone H3.3 variant was very likely ancestral to H3 variants of all opisthokonts. H3.2 and H3.1 as well as H3.1t are derivatives of H3.3, whereas H3.2 evolved already in early branching animals, such as *Trichoplax*. We found no evidence for H3.1 and H3.1t outside of mammals. The earlier observed numerical increase of particular H3 variants towards mammalian evolution [9] is biased, since some "lower" eukaryotes have similar or even higher numbers of H3 variants.

Our study confirms that protoH3 of LECA most probably was rather invariant from stem H3 variants, showing that grouping of divergent H3 variants from mostly parasitic representatives of putatively early branching eukaryotes close to the eukaryotic root is an artifact. These H3 variants represent derived states rather than being ancestral.

At least a basal repertoire of chromatin modifying mechanisms must share a conserved common ancestry and thus be inherent to all eukaryotes, as shown by the presence of various PTMs on H3 tails in selected species with conserved H3 sequences representing putatively early branching eukayotic clades.

## Methods

### Bioinformatical sequence acquisition

Various nucleotide databases (Histone Sequence Database, GeneBank, RefSeq, TBestDB) were scanned for H3 sequences using *Drosophila melanogaster *H3.3 or CenpA protein sequence as query for tBlastn. H3-similar hits were virtually translated into proteins and used for alignment analyses. To identify phylogenetically distant H3 or CenH3 variants from putatively early branching eukaryotic clades we re-used more diverging H3 variants found before in some cases as query sequences for tBlastn. Sequence fragments were assembled to full-length sequences where sufficient fragment overlap was found.

### Telomere suppression PCR and expression profiling

We fully characterized *Stylonychia lemnae *macronuclear genome encoded H3 variants using degenerate oligonucleotides in combination with telomere suppression PCR (TSP), a technique to amplify the 5'- or 3'-ends of *Stylonychia *nanochromosomes including their telomeric sequences [45]. Sexual reproduction of *Stylonychia *was initiated by mixing equal numbers of cells from different mating types. Samples for cDNA synthesis were taken periodically at time points as indicated. Total RNA was isolated as described earlier [46]. Subsequently, cDNA was synthesized using the Qiagen QuantiTect Reverse Transcription kit. Quantitative Real-Time PCR was performed on a Roche Light Cycler.

### Alignments

Aligments were performed using ClustalW included in MEGA 4.1 [47] and were subsequently manually refined.

### Phylogenetic analyses and ancestral state reconstruction

Phylogenetic tree calculations were conducted using MEGA 4.1 [47] software.

The evolutionary history of 159 H3 and CenH3 variants (Figure 2A) was inferred using the Neighbor-Joining method [48]. The bootstrap consensus tree inferred from 1.000 replicates [49] was taken to represent the evolutionary history of the taxa analyzed. Evolutionary distances were computed using the JTT matrix-based method [50] and are in the units of the number of amino acid substitutions per site. All positions containing alignment gaps and missing data were eliminated only in pairwise sequence comparisons. The final dataset contained a total of 100 positions.

The evolutionary relationship of 128 non-redundant histone H3 variants (Figure 2B) was inferred as described above. The bootstrap consensus tree was inferred from 10.000 replicates [49]. Evolutionary distances were computed as described above. The final dataset contained a total of 358 positions.

Ancestral states represented by selected internal nodes from clades well supported by NJ tree topology were reconstructed (compare node markers in Figure 2). The putative ancestral sequences were subsequently inspected by eye and manually refined.

### Immunofluorescence microscopy

Cells were fixed in 2% paraformaldehyde (*Stylonychia*, *Trichomonas*) or alternatively in methanol:acetic acid (3:1) (*Euglena*), washed twice with phosphate buffered saline (PBS), and immobilized onto poly-L-lysine coated coverslips. Subsequently immunostaining with PTM-specific antibodies and in some experiments peptide competition assays were performed as described earlier [29]. Cells were analyzed by confocal laser scanning microscopy (CLSM). Acquisition of serial sections was done with a Zeiss LSM 5 Pascal confocal laser scanning microscope equipped with a water objective lens (Plan-Neofluar 25/0.8, or in some cases C-Apochromat 63/1.2). Fluorochromes were visualized with an argon laser with excitation wavelengths of 488 nm for Alexa Fluor 488 and 514 nm for SYTOX Orange. Fluorochrome images were scanned sequentially generating 8 bit grayscale images. Image resolution was 512 × 512 pixels with variable pixel size depending on the selected zoom factor. The axial distance between light optical serial sections was 300 nm. To obtain an improved signal to noise ratio each section image was averaged from four successive scans. The 8 bit grayscale single channel images were overlaid to an RGB image assigning a false color to each channel and then assembled into tables using open source software ImageJ (Rasband, W.S., ImageJ, National Institutes of Health, Bethesda, Maryland, USA, http://rsb.info.nih.gov/ij/, 1997-2004.) and Adobe Photoshop CS3 software.

### SDS Page and Western Analyses

Cells were lysed, and subsequently total cellular proteins were resuspended in loading buffer [51], heated for 10 min at 100°C, and separated on 15% sodium dodecyl sulfate (SDS)-polyacrylamide gels. Proteins were then transferred onto a nylon membrane and probed with specific antibodies. Detection was done using the digoxigenin system (Roche).

## Authors' contributions

JP designed and coordinated this study and wrote the manuscript. JP further carried out data acquisition, phylogenetic analyses as well as microscopy and Western analyses. SF carried out the characterization of *Stylonychia *H3 variant nanochromosomes and performed expression analyses. WJC participated in the phylogenetic analyses and helped to draft the manuscript. HJL took part in the coordination of the study and helped to draft the manuscript. All authors read and approved the final manuscript.

## Supplementary Material

Additional file 1 **Consensus sequence cartoon of histone H3 (long branching sequences removed) and aligned protein sequences of 128 H3 variants**. Amino acid positions refer to human H3.1. Identical sites are shaded in black, similar residues are shaded in light grey. The positions of four helix motifs within the histone fold domain and the putative chaperone recognition domain are marked at the top of the alignment. Click here for file

Additional file 2 **Phylogenetic tree of eukaryotic life (simplified after **[23]**)**. The position of selected species is highlighted. Click here for file

Additional file 3 **FASTA formatted histone H3 variant sequence alignment**. Click here for file

Additional file 4 **Overview about the most frequent residue alterations in various ancestral state sequences of histone H3 (compare **Figure 3**)**. Variable sites are highlighted (*); symbols beneath list the most frequent amino acid variations. Outgroup taxons used for ancestral state reconstruction a displayed within brackets for each clade. Click here for file

Additional file 5 The alignment contains some exemplary N-terminal chromodomain sequences of putative Hp1-like proteins from putatively early branching eukaryotes, which possess a set of conserved residues (*) formally required for ARKme3S/T binding. Residues identical in 85% of all sequences are shaded black; residues similar in 85% of all sequences are shaded grey. Notably, in three *Trichomonas vaginalis *sequences a C-terminal chromoshadowdomain could be recognized (C+CS). Click here for file
