# Supplementary figures and images for: The evolutionary history of histone H3 suggests a deep eukaryotic root of chromatin modifying mechanisms
Source: BMC Evol Biol. 2010 Aug 25;10:259. doi: 10.1186/1471-2148-10-259 (PMC2939574; doi:10.1186/1471-2148-10-259)

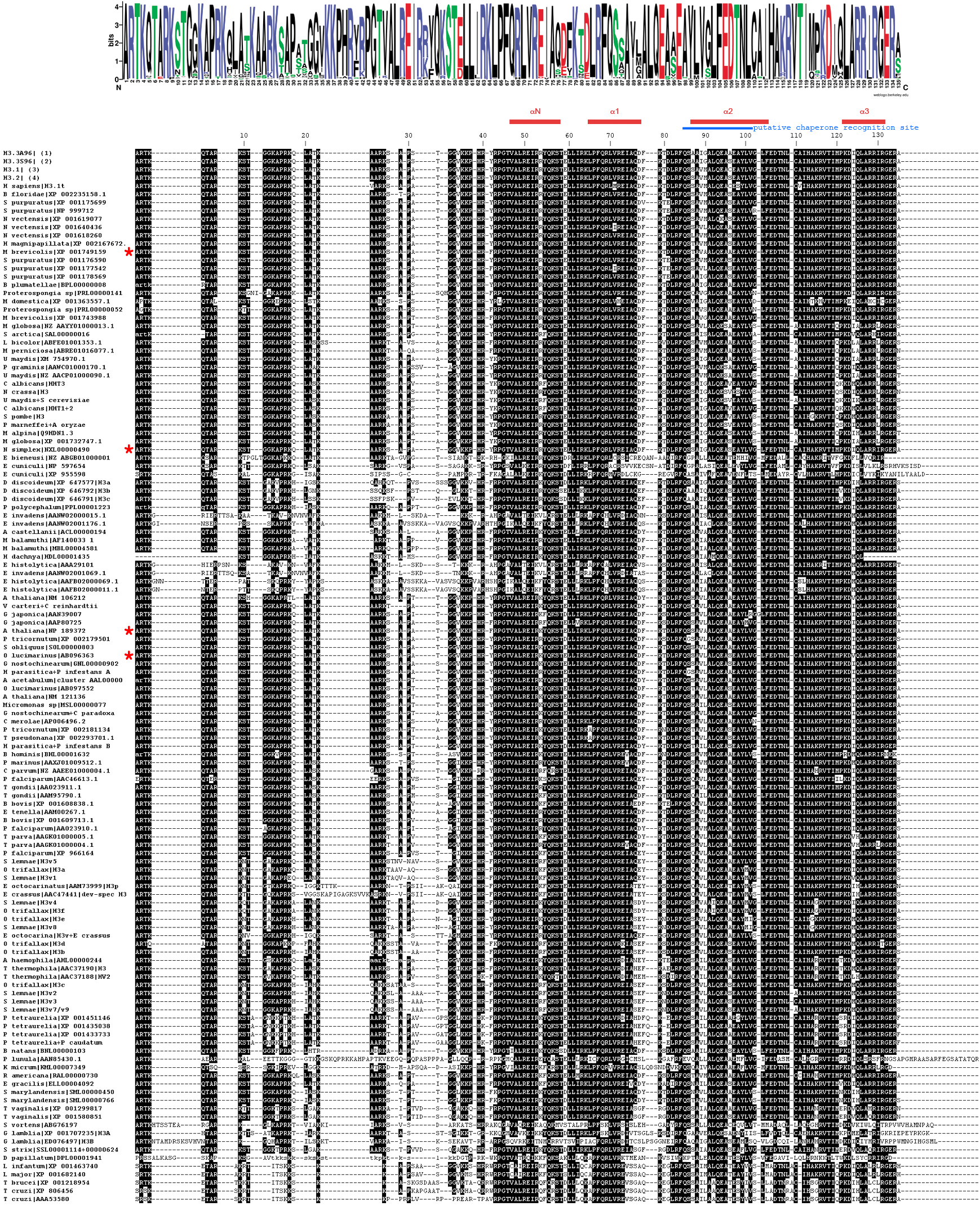

Supplement: Additional file 1 — Consensus sequence cartoon of histone H3 (long branching sequences removed) and aligned protein sequences of 128 H3 variants. Amino acid positions refer to human H3.1. Identical sites are shaded in black, similar residues are shaded in light grey. The positions of four helix motifs within the histone fold domain and the putative chaperone recognition domain are marked at the top of the alignment. [file 1471-2148-10-259-S1.JPEG]

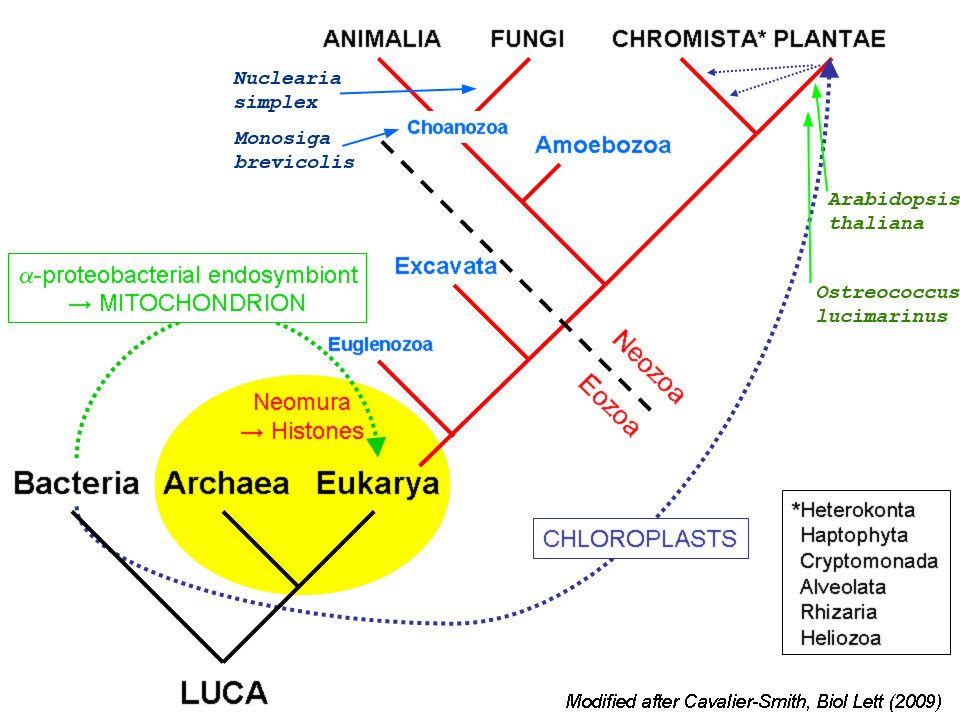

Supplement: Additional file 2 — Phylogenetic tree of eukaryotic life (simplified after [23]). The position of selected species is highlighted. [file 1471-2148-10-259-S2.JPEG]

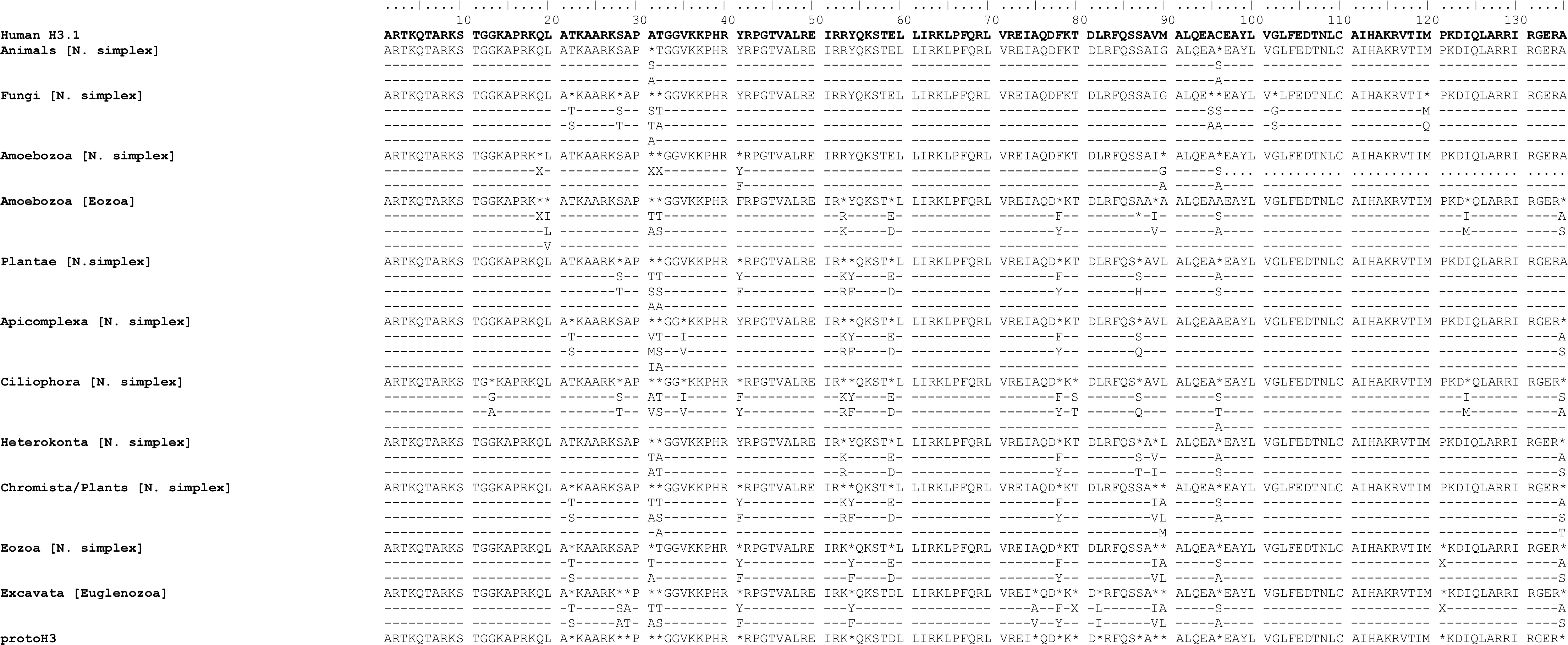

Supplement: Additional file 4 — Overview about the most frequent residue alterations in various ancestral state sequences of histone H3 (compare Figure 3). Variable sites are highlighted (*); symbols beneath list the most frequent amino acid variations. Outgroup taxons used for ancestral state reconstruction a displayed within brackets for each clade. [file 1471-2148-10-259-S4.JPEG]

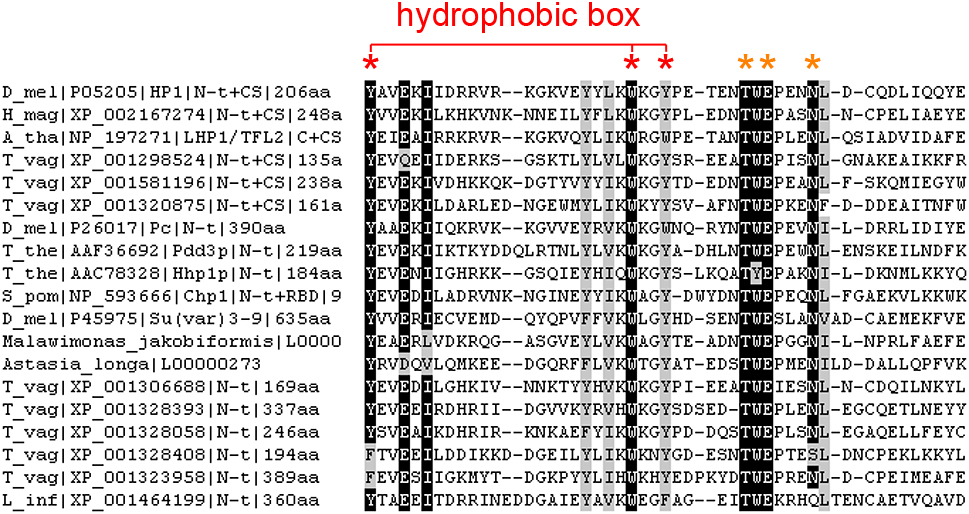

Supplement: Additional file 5 — The alignment contains some exemplary N-terminal chromodomain sequences of putative Hp1-like proteins from putatively early branching eukaryotes, which possess a set of conserved residues (*) formally required for ARKme3S/T binding. Residues identical in 85% of all sequences are shaded black; residues similar in 85% of all sequences are shaded grey. Notably, in three Trichomonas vaginalis sequences a C-terminal chromoshadowdomain could be recognized (C+CS). [file 1471-2148-10-259-S5.JPEG]
